# Desmoid tumor occurrence following gastric cancer surgery: A report of two cases

**DOI:** 10.1016/j.ijscr.2023.108824

**Published:** 2023-09-20

**Authors:** Masumi Takamoto, Noriyuki Nishiwaki, Yoshihiro Mikuriya, Tomokazu Kakishita, Koji Ohta, Shinji Hato

**Affiliations:** Department of Gastroenterological Surgery, National Hospital Organization, Shikoku Cancer Center, 160, Ko, Minamiumemoto-machi, Matsuyama-shi, Ehime-Ken 791-0280, Japan

**Keywords:** Desmoid, Gastric cancer, Surgical resection

## Abstract

**Introduction and importance:**

While rare, desmoid tumors can develop after abdominal surgery and are difficult to differentiate from recurrent tumors following cancer resection. In this report, we describe two cases of desmoid tumors that occurred following gastric cancer procedures and were successfully treated with surgical resection.

**Case presentation:**

In Case 1, a 77-year-old woman underwent open distal gastrectomy for gastric cancer followed by Roux-en-Y reconstruction. The pathological diagnosis was stage IIB T3N1M0 disease. Four years postsurgically, computed tomography (CT) revealed a 2.4 cm tumor lesion in the upper abdomen. Desmoid tumor was the most suspected tumor, for which a resection with partial resection of the jejunum was performed.

In case 2, a 60-year-old man underwent open distal gastrectomy for gastric cancer and Billroth I reconstruction; the pathological diagnosis was T1aN0M0 stage IA. Two years later, CT revealed a 4.0 cm tumor lesion in the upper abdomen. As in Case 1, desmoid tumor was most suspected, a tumor resection with partial resection of the jejunum was performed.

Based on the pathological findings, the tumors were diagnosed as desmoid tumor. There had been no recurrence of either gastric cancer or the desmoid tumor in both cases.

**Clinical discussion:**

Although active surveillance has been recommended for desmoid tumors recently, surgical resection is appropriate when recurrence cannot be ruled out.

**Conclusions:**

Desmoid tumors should be included in the differential diagnosis when intra-abdominal tumors occur after surgery for gastric cancer. Complete resection with adequate margins can prevent desmoid recurrence.

## Highlights

• While rare, desmoid tumors can develop after gastric cancer surgery
• Wnt/ β-catenin pathway plays an important role in desmoid formation after surgery.
• Recently, active surveillance has been recommended for desmoid tumors.
• Surgical resection is appropriate when recurrence cannot be ruled out.
• Complete resection with adequate margins can prevent desmoid recurrence.

## 1 Introduction

Desmoid tumors are non-cancerous growths that occur in connective tissue, often in the abdomen, arms, or legs. They are constituting <3 % of soft tissue neoplasms with an estimated annual incidence of three to five cases per million worldwide. Desmoid tumors occur predominantly in women, and the risk of desmoid tumor development appears to increase during and after pregnancy. Most desmoid tumors occur sporadically but about 2–5 % commonly occur in the abdominal; cavity or abdominal wall in association with familial adenomatous polyposis (FAP). They can occur after abdominal surgery and are difficult to distinguish from recurrence after cancer procedures, though this is rare; the reported incidence of gastric cancer following surgery is only approximately 0.05 % [1]. Here, we describe two cases of desmoid tumors that were successfully treated with surgical resection after gastric cancer surgery. This case report has been reported in line with the SCARE Criteria [2].

## 2 Case presentation

### 2.1 Case 1

**Fig. 1:**
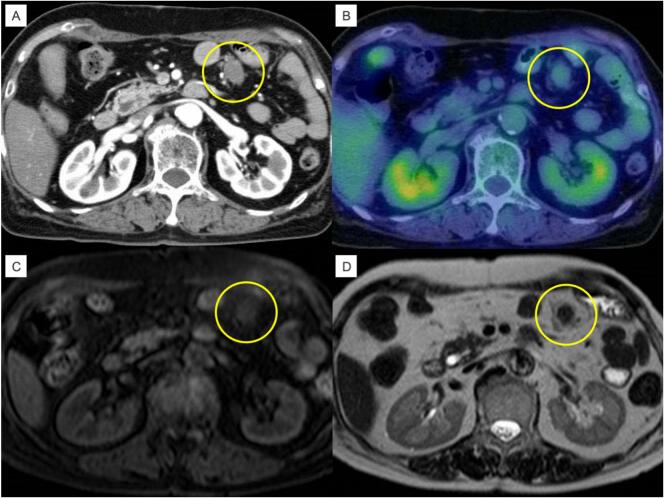
Preoperative image. CT showed a soft-tissue tumor in the mesentery of the jejunum (yellow area) (a). PET-CT showed the presence of an area with pale accumulation of FDG (b). T2-weighted MRI showed a low signal (c). T1-weighted MRI showed an equal signal (d). (For interpretation of the references to colour in this figure legend, the reader is referred to the web version of this article.)

**Fig. 2:**
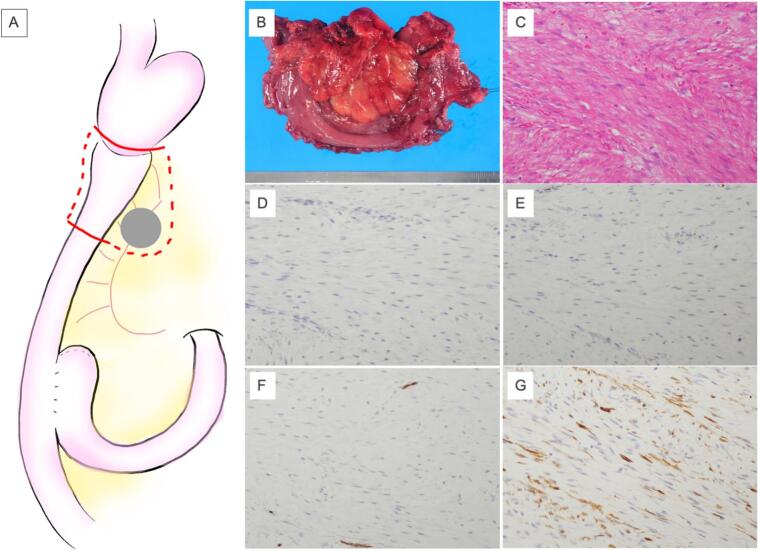
Intraoperative findings. Surgical schema (a). Macroscopic and microscopic findings of the tumor, immunostaining findings of the tumor. The tumor was located in the mesentery of the jejunum. The resected specimen was a 2.8 cm × 2.0 cm, nodular mass (b). There was an intricate proliferation of spindle-shaped cells with collagen fibers (c). SM-actin was partially positive (d). Desmin was negative (e). S100 was negative (f). CD34 was negative (g).

A 77-year-old woman underwent open distal gastrectomy and Roux-en-Y (RY) reconstruction for gastric cancer. The pathological diagnosis was T3N1M0 stage IIB (Japanese classification of gastric cancer 14th), and the patient was a candidate for adjuvant chemotherapy according to the guidelines [3]. However, she was unable to continue the therapy because of abdominal pain, dizziness, and anorexia. Computed tomography (CT) performed 4 years after gastric cancer surgery revealed a 2.4 cm large tumor lesion in the upper abdomen (Fig. 1A). Positron emission tomography (PET) showed pale ^18^F-fluorodeoxyglucose (FDG) accumulation (standardized uptake value (SUV)_max_ = 1.98) in the tumor. Abdominal magnetic resonance imaging (MRI) revealed that the soft tissue mass in the jejunal mesentery showed a low signal on T2-weighted images and an equal signal on T1-weighted images (Fig. 1B, C, and D). Retrospective CT evaluation of the tumor revealed a slow increase in size over the course of approximately three years. She had no family history of FAP, and no other abnormalities were observed. Although the possibility of recurrence could not be ruled out due to the patient's history of gastric cancer, based on imaging findings and the speed of tumor growth, a desmoid tumor was the most likely suspect, and GIST was also considered as a differential diagnosis. The patient underwent surgery for diagnostic and therapeutic purposes. Intraoperative findings showed that the tumor was located close to the gastrojejunostomy site in the mesentery of the jejunum and had invaded the marginal artery. Partial jejunal tumor resection was then performed (Fig. 2A), revealing that the tumor was 2.8 × 2.0 cm in size. Hematoxylin-eosin (HE) staining showed an intricate proliferation of spindle-shaped cells with collagen fibers (Fig. 2B). Cellular dysplasia was mild, with a fission picture of <1 cell/ 10 high power fields (HPF). Immunohistochemistry (IHC) staining was negative for desmin, S100, and CD34 (Fig. 2D, E, F) and partially positive for smooth muscle actin (Fig. 2G). Based on these findings, the tumor was diagnosed as desmoid. The patient was then discharged on the 40th postoperative day due to postoperative paralytic ileus. Seven years after desmoid tumor resection, there had been no recurrence of gastric cancer or desmoid tumors.

### 2.2 Case 2

**Fig. 3:**
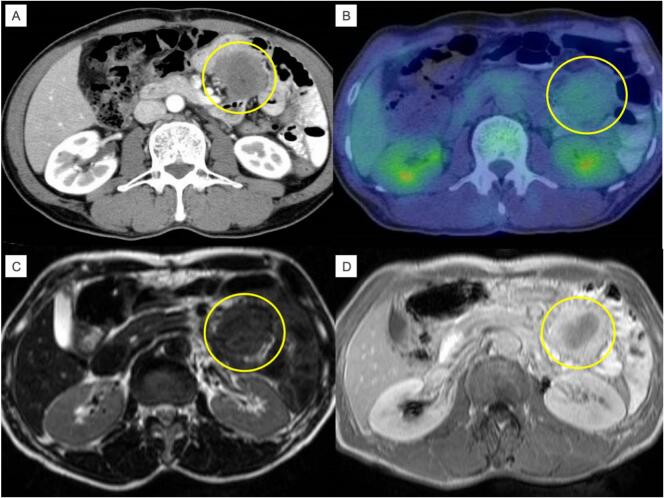
Preoperative image. CT showed a soft-tissue tumor in the mesentery of the jejunum (yellow area) (a). PET-CT showed the presence of an area with pale accumulation of FDG (b). T2-weighted MRI showed a low signal (c). T1-weighted MRI showed a low signal (d). (For interpretation of the references to colour in this figure legend, the reader is referred to the web version of this article.)

**Fig. 4:**
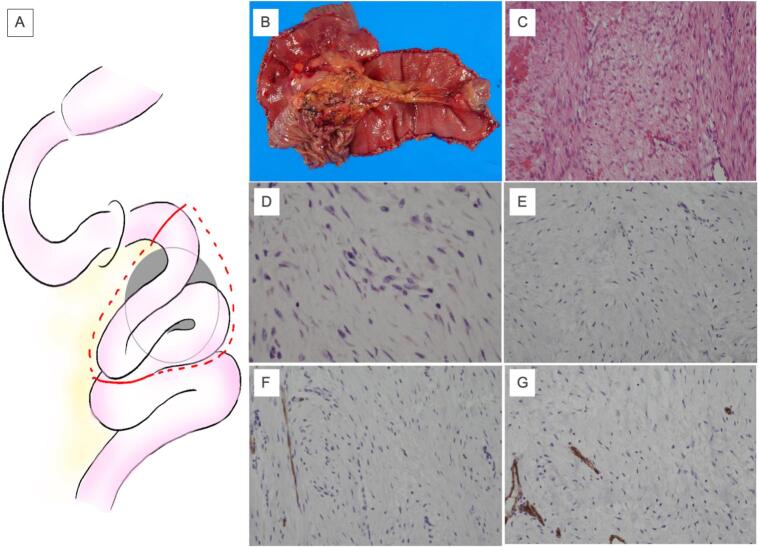
Intraoperative findings. Surgical schema (a). Macroscopic and microscopic findings of the tumor, immunostaining findings of the tumor. The tumor was located in the mesentery of the jejunum. The resected specimen was a 5.5 cm × 6.5 cm, nodular mass (b). There was intricate proliferation of spindle-shaped cells with collagen fibers (c). Catenin was negative (d). Desmin was negative (e). S100 was negative (f). CD34 was negative (g).

A 60-year-old man underwent open distal gastrectomy for gastric cancer and Billroth I reconstruction. The pathological diagnosis was T1aN0M0 stage IA (Japanese Classification of Gastric Cancer 13th), and adjuvant chemotherapy was not administered according to the guidelines [3]. A 4.0 cm tumor was then found in the upper abdomen on a CT scan performed in investigation of recurrent epigastric pain two years after gastric cancer surgery to investigate recurrent epigastric pain (Fig. 3A). PET revealed slight FDG accumulation (SUV_max_ = 2.46) in the tumor. MRI of the abdomen revealed that a soft tissue mass in the mesentery of the jejunum showing a low signal on both T2-weighted and T1-weighted images (Fig. 3B, C, D). Retrospective CT showed that the tumor had grown rapidly over the course of approximately one year. He had no family history of FAP, and no other abnormalities were observed. Although the possibility of recurrence and another malignant tumor type, gastrointestinal stromal tumor (GIST), could not be ruled out due to the rapid growth, desmoid tumor was most suspected based on imaging findings. The patient then underwent surgery for diagnostic and therapeutic purposes, and intraoperative findings showed that the tumor was located in the mesentery of the jejunum and had invaded the marginal arteries. Partial jejunal tumor resection was then performed (Fig. 4A). The tumor was 5.5 × 6.5 cm in size. HE staining showed intricate proliferation of spindle-shaped cells with collagen fibers (Fig. 4B). Cellular dysplasia was considered; however, no increase was observed in the fission images. IHC staining was negative for catenin, desmin, S100, and CD34 (Fig. 4C, D, E, F, and G). Based on these findings, the tumor was diagnosed as desmoid. There were no postoperative complications, and the patient was discharged on the 12th postoperative day. Twelve years after resection of the desmoid tumor, there had been no recurrence of either gastric cancer or the desmoid tumor.

## 3 Discussion

This study described two cases of desmoid tumors that occurred following gastric cancer surgery and were successfully treated with surgical resection. Although it was difficult to distinguish between the recurrence of gastric cancer and desmoid tumors on preoperative imaging, tumor resection with partial resection of the jejunum was performed, and no recurrence followed after a considerable follow-up period.

Desmoid tumors are a type of fibromatosis that are considered borderline malignant as they are histologically benign but show local recurrence [4]. Although their etiology is unknown, most desmoid tumor is associated with alterations in the Wnt/β-catenin pathway. In 85–90 % sporadic cases have a mutation in the CTNNB1 encoding β -catenin. In patients with FAP, desmoid tumors harbor germ line mutations in the adenomatous polyposis coli (APC) gene, which encodes for a protein regulating β -catenin levels. We also acknowledge the involvement of external factors such as trauma or after abdominal surgery and internal factors such as female hormones. However, few reports on association with adjuvant chemotherapy. Since no genetic mutations associated with gastric cancer have been reported to be associated with desmoid tumors, surgical trauma may play a role in desmoid tumor development in postoperative cases of gastric cancer. Inflammatory trauma induces β -catenin up-regulation and proliferation of clonal myofibroblasts, leading to desmoid tumor formation. Desmoid tumors occur in the connective tissue and often in the abdominal wall (49 %), extra-abdominal wall (43 %), and intra-abdominal (8 %) [5]. Imaging diagnosis of these tumors is often difficult because of the lack of typical findings [6]; PET shows only a mild accumulation of FDG, and the contrast effect of CT is variable. In these cases, because there were no specific findings on the images, it was difficult to differentiate the tumor from gastric cancer recurrence. In addition, desmoid tumors have no specific symptoms and early detection is difficult. Intra-abdominal desmoid tumors may invade nearby organs, resulting in bowel obstruction, bowel perforation, and urinary retention. Ultimately, we decided to perform tumor resection because of the lack of FDG accumulation, speed of tumor growth, and tumor localization, suggesting a desmoid tumor.

**Fig. 5:**
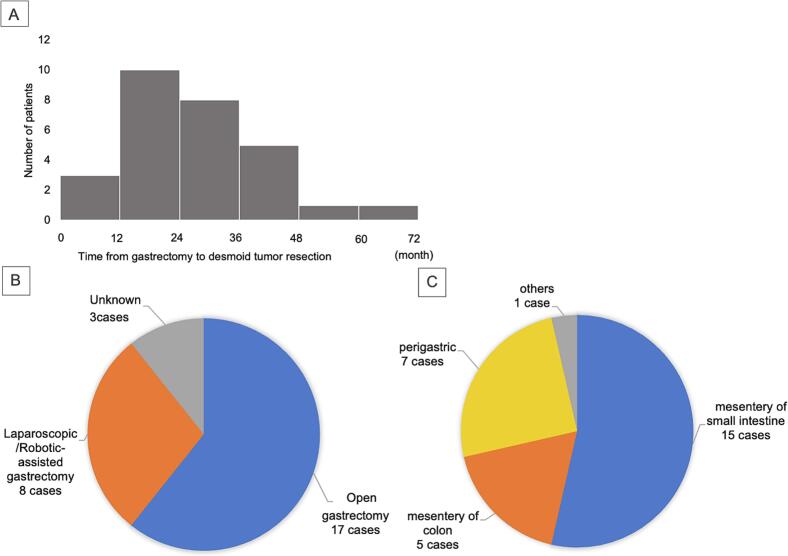
Characteristics of 28 patients with intra-abdominal desmoid tumors after gastric cancer surgery are shown. Time after gastric cancer surgery to desmoid tumor resection (a). Surgical procedures for gastric cancer (b). Location of desmoid tumors (c).

The occurrence of desmoid tumors after gastrectomy is extremely rare. Takeuchi et al. reported an incidence of 0.05 % in a review of 1942 gastric cancer gastrectomy cases [1]. To the best of our knowledge, there have been 28 reported cases of desmoid tumors arising after gastric cancer surgery, including our two cases (Table 1) (Fig. 5) [1,[7], [8], [9], [10], [11], [12], [13], [14], [15], [16], [17], [18], [19]]. Of these, the median age of patients was 62 years, 24 (85 %) patients were male, the median tumor diameter was 5 cm, and the median time from gastrectomy to desmoid tumor resection was 25 months. Open gastrectomy was performed in 17 cases (60 %), and the tumor location was predominantly in the upper abdomen, in the mesentery of the small intestine (15 cases), around the remnant stomach (seven cases), or in the mesentery of the transverse colon and ileum (five cases), consistent with previous reports of a high incidence of surgical operation sites [20]. In our cases, Case 1 was diagnosed four years following gastrectomy, whereas in Case 2 was two years, and the tumor location was the mesentery of the jejunum, in agreement with previously reported cases regarding timing and location of desmoid tumors after gastrectomy.

**Table 1:** Reported cases of desmoid tumor after gastrectomy for gastric cancer.

Variables			Reference
Age			
Median (range)	62	44–88	
Sex			
Male	24	85.7 %	1,6-12,15-22,24–29
Female	4	14.3 %	14, 23
Pathological stage			
I	18	64.3 %	1,6,8,10-12,14, 17,19,21,23,26, 27, 29
II	4	14.3 %	9, 16, 18, 28
Unknown	6	21.4 %	7,15, 20, 22, 24, 25
Gastrectomy			
Open gastrectomy	17	60.7 %	11, 12, 14, 15, 17, 19–25, 27,28
Laparoscopic/Robotic-assisted gastrectomy	8	28.6 %	1, 6, 9, 10, 16, 18, 29
Unknown	3	10.7 %	7, 8, 26
Interval between gastrectomy and desmoid resection (months)			
Median (range)	25	12–72	
Tumor size(cm)			
Median (range)	5	1.3–11.5	
Tumor location			
Mesentery of small intestine	15	53.5 %	1, 6, 11, 16, 17, 19–22, 24, 25, 28, 29
Mesentery of colon	5	17.8 %	7, 8, 12, 23, 27
Perigastric	7	25.0 %	6, 9, 10, 14, 15, 18
Others	1	3.5 %	26
Preoperative diagnosis (duplicate)			
Recurrence of gastric cancer	16	–	1, 7, 9–12, 14, 16–18, 22, 25, 27
Desmoid tumor	6	–	8, 10, 26, 28
Mesenteric tumor	5	–	11, 16, 19, 20, 21
GIST	5	–	6, 10, 27, 29
Non-epithelial malignant tumor	2	–	7, 23
Others	10	–	1, 6, 8, 10, 15, 24, 27
Combined resection			
Small intestine	12	36.4 %	1, 6, 11, 19, 20, 22, 25, 26, 28, 29
Large intestine	8	24.2 %	6–9, 12, 17, 23, 27
Remnants of a stomach	3	9.1 %	10, 15, 18
Others	10	30.3 %	6, 7, 10, 14–16, 18, 21, 24
Recurrence			
None	20	71.4 %	1, 6, 8–12, 14, 15, 17, 19, 21, 22, 27–29
Unknown	8	28.6 %	7, 16, 18, 20, 23–26

Recently, active surveillance has been recommended as a treatment policy for desmoid tumors [21,22]. According to the Desmoid Tumor Working Group, systemic control strategies should be implemented when the tumor has progressed during surveillance [23]. Anti-inflammatory drugs, cytotoxic chemotherapy, tyrosine kinase inhibitors, and γ-secretase inhibitors are newer and more evidence-based therapeutic options, while hormone therapies are no longer routinely recommended. Considering the relatively high postsurgical local recurrence rates, surgery is no longer considered the primary therapy for patients with desmoid tumor. On the other hand, radiation therapy alone may provide local control in patients with progressive disease, for whom surgery is not an option and who are not otherwise controlled with medical therapy.

Although novel therapeutic approaches are being explored, some patients still require surgery. The preoperative imaging diagnosis of desmoid tumors is difficult. According to previous reports, only six patients (21 %) underwent surgery with a diagnosis of desmoid tumors, while 22 patients (79 %) were considered to have malignant tumors. Therefore, surgical resection is appropriate when the possibility of recurrence or other diseases cannot be ruled out, as in the present case. Although we did not attempt laparoscopic surgery since these two cases underwent open gastrectomy, laparoscopic surgery for desmoid tumor may allow a more minimally invasive procedure. Despite the possibility of publication bias, complete resection with adequate margins may effectively prevent recurrence; no recurrence has been reported among the 28 previously reported cases, excluding eight unknown cases. Desmoid tumors causing impaired gastrointestinal transit may also require surgery. While adequate margins are necessary to prevent recurrence, the extent of resection must be carefully considered to preserve organ function.

## 4 Conclusions

In addition to presenting two cases of desmoid tumors that were successfully treated with surgical resection, this study provides a literature review of desmoid tumors after gastric cancer surgery. Desmoid tumors should be considered in the differential diagnosis of intra-abdominal tumors after gastric cancer surgery. When surgically removing tumors suspected to be desmoid, complete resection with adequate margins can prevent recurrence.

## List of abbreviations

APC: adenomatous polyposis coli
CT: computed tomography
FDG: ^18^F-fluorodeoxyglucose
FAP: familial adenomatous polyposis
GIST: gastrointestinal stromal tumor
HE: Hematoxylin-Eosin
HPF: high power fields
IHC: Immunohistochemistry
MRI: magnetic resonance imaging
PET: positron emission tomography
SUV: standardized uptake value
RY: Roux-en Y

## Consent for publication

Written informed consent was obtained from the patient for the publication of this case report and any accompanying images. A copy of the written consent form is available for review by the editor-in-chief of the journal.

## Guarantor

Noriyuki Nishiwaki.

## Registration of research studies

This is not First in Man case report.

## Ethics approval and consent to participate

Not applicable.

## Funding

This study did not receive any funding.

## CRediT authorship contribution statement

MT drafted the manuscript and provided the original images. SH performed all surgeries. NN, TK, and SH reviewed and revised the manuscript. All the authors have read and approved the final version of the manuscript.

## Declaration of competing interest

The authors have no financial or personal circumstances with pharmacists or organizations that could influence the originality of this manuscript.

## Data Availability

The data supporting these conclusions are included in this article.
